# Actinobacteria associated with *Glycyrrhiza inflata* Bat. are diverse and have plant growth promoting and antimicrobial activity

**DOI:** 10.1038/s41598-018-32097-8

**Published:** 2018-09-12

**Authors:** Ke Zhao, Jing Li, Xiaoyue Zhang, Qiang Chen, Maoke Liu, Xiaolin Ao, Yunfu Gu, Decong Liao, Kaiwei Xu, Monggeng Ma, Xiumei Yu, Quanju Xiang, Ji Chen, Xiaoping Zhang, Petri Penttinen

**Affiliations:** 1 Department of Microbiology, College of Resource and Environmental Sciences, Sichuan Agricultural University, Yaan, 625000 P. R. China; 2 Biotechnology Center, Rice and Sorghum Research Institute, Sichuan Academy of Agricultural Sciences, Luzhou, 646100 P. R. China; 3 Zhejiang Provincial Key Laboratory of Carbon Cycling in Forest Ecosystems and Carbon Sequestration, School of Environmental & Resource Sciences, Zhejiang Agriculture & Forestry University, Linan, 311300 P. R. China; 4 Ecosystems and Environment Research Programme, University of Helsinki, Helsinki, Fin-00014 Finland

## Abstract

Many of the plant associated microbes may directly and indirectly contribute to plant growth and stress resistance. Our aim was to assess the plant growth-promoting and antimicrobial activities of actinobacteria isolated from *Glycyrrhiza inflata* Bat. plants to find strains that could be applied in agricultural industry, for example in reclaiming saline soils. We isolated 36 and 52 strains that showed morphological characteristics of actinobacteria from one year old and three year old *G*. *inflata* plants, respectively. Based on 16S rRNA gene sequence analysis, the strains represented ten actinobacterial genera. Most of the strains had plant growth promoting characteristics *in vitro*, tolerated 200 mM NaCl and inhibited the growth of at least one indicator organism. The eight selected *Streptomyces* strains increased the germination rate of *G*. *inflata* seeds under salt stress. In addition, the four best seed germination promoters promoted the growth of *G*. *inflata in vivo*. The best promoters of *G*. *inflata* growth, strains SCAU5283 and SCAU5215, inhibited a wide range of indicator organisms, and may thus be considered as promising candidates to be applied in inoculating *G*. *inflata*.

## Introduction

Plants offer diverse habitats for numerous microorganisms. Parasites, mutualists and commensals live in plant rhizosphere, inside plant tissues as endophytes, and on the surface of the aerial organs. The plant-associated habitats provide resident microorganisms with nutrients and energy, which is likely to apply a selection pressure on the microorganisms. Microbes in the rhizosphere benefit from exudation of organic compounds from roots and from dead root material, microbes living on above ground plant surfaces can benefit from nutrients leaching from plant tissues, and microbes living inside plants can access nutrients directly^1^. Abiotic factors, including water availability, temperature and solar radiation may directly affect the growth and activities of microorganisms in these habitats. Many of the plant associated microbes may directly and indirectly contribute to plant growth and stress resistance by various mechanisms, including increased availability of minerals, nitrogen fixation, and production of hydrolytic enzymes and phytohormones.

Actinobacteria, Gram-positive bacteria with a high genomic G + C content, are widespread environmental organisms and found in both terrestrial and aquatic habitats. The diversity of ecologically important plant associated actinobacteria is significantly affected by plant tissue type and growth stage, and soil nutrient availability^2–5^. Plant-associated actinobacteria may affect plant growth and improve the stress resistance of their host plants^6–9^. In addition, plant associated actinobacteria are a potential source of novel bioactive metabolites^10,11^. Many plant associated actinobacteria produce antifungal or antibacterial agents, for example extracellular hydrolytic enzymes that lyse fungal cell walls^12^.

Liquorice (*Glycyrrhiza* spp.) is one of the most ancient herbal medicines. The root and rhizomes of *Glycyrrhiza inflata* Bat., *Glycyrrhiza glabra* L. and *Glycyrrhiza uralensis* Fisch. have been widely used as a flavoring agent and for a variety of pharmaceutical applications for thousands of years in southern Europe and parts of Asia^13^. *G*. *inflata* is found mainly in Xinjiang, China^14^. It is one of the main sources of liquorice in Chinese medicine due to the presence of a wide variety active ingredients, for example triterpenoids, flavonoids, and polysaccharides^15,16^. *Glycyrrhiza* spp. belong to Leguminosae, and they are nodulated by diverse rhizobia with plant growth promoting (PGP) activity^17–19^. Generally, liquorice grow in Central Asia, Mongolia, Iraq and the northwest of China, in regions characterized by harsh environmental conditions, including high temperatures and evaporation, high salinity, low precipitation, poor soil condition, and strong winds and UV irradiation. Liquorice plants have been applied to remediate saline soils^20^. Inoculation with plant growth promoting (PGP) bacteria may benefit the remediation process, since they can increase germination and seedling growth in saline conditions^21,22^.

In our previous study we characterized the diversity and antimicrobial activity of actinobacterial isolated from *G*. *inflata* and *G*. *glabra*^23^. However, to our knowledge the plant growth promoting activity of actinobacteria associated with *Glycyrrhiza* spp. has not been studied. Therefore, our aim was to assess the plant growth-promoting properties, salt tolerance and antimicrobial activities of actinobacteria isolated from *G*. *inflata* to find strains that could be applied in agricultural industry, for example in reclaiming saline soils. Since the endophytic communities in different plant organs may differ and change during plant growth^24^, we sampled bark, leaf, root, and stem from both young and mature plants to increase our possibilities to isolate strains with desired characteristics.

## Materials and Methods

### Sample collection

Healthy one year old and three year old *Glycyrrhiza inflata* Bat. plants were randomly collected from Tarim in Xinjiang, China. Plants were sampled in triplicates. The sampling area is arid desert characterized with low rainfall and high evaporation. The soil is classified as sandy soil. The plants were dug out and bulk soil was removed by gentle shaking. Plants were kept at 4 °C, brought to the laboratory and processed immediately. The bark, leaf, root, and stem were separated and surface sterilized as described previously^25^. Aliquots from the final rinse were incubated on ISP2 media at 28 °C for 3–4 weeks. The sterilization was regarded effective when there was no growth.

### Isolation and preliminary identification of endophytic actinobacteria

Surface-sterilized plant samples were aseptically cut into small fragments using commercial blender. Subsequently, the fragments were plated onto five selective isolation media: Tap Water Yeast Extract Agar (TWYE)^26^, Starch Casein Nitrate Agar (SCNA), Chitin Agar, Humic-vitamine Agar (HV)^27^, and Oatmeal Agar (ISP_3_). Isolation media were supplemented with nalidixic acid and K_2_Cr_2_O_7_ (50 µg ml^−1^) to inhibit the growth of non-actinobacteria. Purified isolates were stored on ISP_4_ slope medium at 4 °C.

The isolates were preliminarily identified by cultural and morphological characteristics as described previously^25^ using light microscopy (Olympus CX31, Olympus Corp., Japan) to observe the spore chain morphology of isolates grown for 10 d on ISP_4_ media.

### DNA extraction, PCR amplification and DGGE analysis

DNA was extracted from 100 mg of fresh tissue with Power Plant^TM^ pro DNA Isolation Kit (MO BIO Laboratories, Carlsbad, CA) according to the manufacturer’s instructions. Extracts were stored at −20 °C. In the first round of a nested PCR 16S rRNA gene was amplified using the primers 243F (5′-GGATGAGCCCGCGGCCTA-3′)^28^ and 1186R (5′-CTTCCTCCGAGTTGACCC-3′)^29^ in a PCR mixture containing 10 µl MIX buffer (Premix Taq^TM^, TaKaRa, China), 1 µl template DNA, 1 µM each primer, and sterile distilled water to the final volume of 20 µl. In the second round a fragment was amplified using the primers 907F (5′-AAACTCAAAGGAATTGACGG-3′)^30^ with a GC-clamp and 1186 R in a PCR mixture containing 25 µl MIX buffer, 1 µl of the first PCR product as template, 1 µM each primer, and sterile distilled water to the final volume of 50 µl. The touchdown PCR was conducted as described previously^31^. Amplification of the approximately 270 bp target fragment was verified by electrophoresis in 2% agarose gel.

PCR products were loaded onto a 8% (w/v) polyacrylamide gel with a 30–60% denaturant gradient in Tris acetate EDTA^32^ buffer and separated for 8 h at 60 °C and 160 V using a Dcode Universal Mutation Detection System (Bio-Rad, USA). After electrophoresis, the gels were silver stained as described earlier^33^. Gel images were acquired using a Gel Doc imaging system (Bio-Rad) and analyzed using Quantity One version™ software. The predominant DGGE bands were excised and reamplified and sequenced at Suzhou GENEWIZ Biological Technology Co., Ltd. (Suzhou, China). The sequences were compared with sequences in the NCBI Genbank nucleotide database using BLASTN to find the closest matching sequences.

### RFLP, sequencing, and phylogenetic analysis of cultivable actinobacteria

Genomic DNA was extracted and purified as described earlier^34^. The 16S rRNA genes were amplified with forward primer 27F (5′-CAGAGTTTGATCCT GGCT-3′) and reverse primer 1492R (5′-AGGAGGTGAT CCAGCCGCA-3′)^35^. The PCR products were digested with restriction endonucleases *Hha*I (TaKaRa, China) for 2 h. The digested fragments were separated in a 2% agarose gel by electrophoresis for 3 h at 60 V and visualized with an UV transilluminator. Isolates were grouped based on the restriction fragment patterns^36^. A phylogenetic tree was constructed using the Unweighted Pair Group with Arithmetic Mean (UPGMA) method in NTSYS 2.1 software^37^. Representative isolates were chosen for 16S rRNA gene sequencing in Suzhou GENEWIZ Biological Technology Co., Ltd. (Suzhou, China). Sequences were compared with NCBI GenBank database using BlastN to find the closest matching sequences. The sequences were pairwise aligned using Clustal X^38^. A phylogenetic tree was constructed under the Kimura two parameter model and bootstrap analyses with 1,000 resamplings using MEGA 6.0^39^.

### Physiological characteristics of the representative strains

Production of indole-3-acetic acid (IAA) and siderophore secretion were assessed as described earlier^40^^,^^41^. The phosphate solubilizing ability was evaluated by using insoluble Ca_3_(PO_4_)_2_ as sole P source in Pikovskaya’s medium^42^. Chitinase activity was estimated as recommended by Xiang *et al*.^43^. Salt resistance was tested by growing the isolates in ISP_4_ media with 0 mM, 100 mM, 200 mM, 300 mM, 400 mM, and 500 mM NaCl at 30 °C for 10 days.

### Evaluation of antimicrobial activity

Representative isolates were tested for their antagonistic activity against seven indicator organisms: *Mycogone perniciosa* Magn [SCAU3216], *Curvularia lunata* Boedijn [SCAU3697], *Alternaria alternata* (Fries) Keissler [SCAU3471], *Fusarium graminearum* Sehw. [SCAU3741], *Fusarium oxysporum* [SCAU3221], *Staphylococcus aureus* [ATCC 25923], and *Escherichia*. *coli* [ATCC35218]. The antagonism was measured as the distance from the mycelium edge to the margin of actinobacterial colony. All strains were tested in triplicates.

### Plant growth promotion assay

Eight strains that were resistant to 400 mM NaCl and produced IAA were selected to study their effects on seed germination under salt stress. To obtain enough spores, the pure cultures were spread on ISP_4_ agar plates and incubated for 5–6 days, after which the agar medium was cut into small pieces. The pieces were transferred on sterilized wheat grains, and incubated at 28 °C until the grains were completely covered with mycelia and spores. The spores were washed off the grains by sterilized distilled water to make a final concentration of 1.0 × 10^8^ CFU mL^−1^ as described previously^2^. *G*. *inflata* seeds were surface sterilized in 1% HgCl (v/w) for 10 min, rinsed three times in sterile distilled water, and inoculated by soaking into the spore suspension for 8 h. Negative control seeds were soaked into sterile distilled water. Seeds were transferred aseptically on MS medium with 0 mM, 100 mM, 200 mM, 300 mM, and 400 mM NaCl with 30 seeds per plate. Treatments were done in three replicates. Germination rate was calculated after 5 day incubation at 28 °C.

Based on the results of the germination test, the isolates SCAU5283, SCAU5276, SCAU5201 and SCAU5207 were selected to test their plant growth promotion activity on *G*. *inflata*. The spore suspension and *G*. *inflata* seeds were prepared as above. *G*. *inflata* seeds were germinated on MS medium with 200 mM at 28 °C. After 3–5 days germination, three seedlings were planted into polypropylene cup filled with a sterilized mixture of washed sand, vermiculite, and ceramic gravel. The surface was covered with 1–2 cm sterilized quartz sand. Cups were put on glass jars filled with sterilized Hoagland’s solution^44^ supplemented with 200 mM NaCl. The seedlings were inoculated with 50 µl of spore suspension around the seedling root. Negative control seedlings were inoculated with 50 µl of sterile distilled water. The treatments were done in three replicates. Seedlings were grown for 45 days in an illuminating incubator using 18 h light period and 6 h dark period at 24 °C and 16 °C, respectively. After harvest, the dry weight, shoot and root length, and N, P, and K contents were measured to evaluate the effect of strains on plant growth. Total N, P, and K contents were determined as described by Liu *et al*.^45^.

### Statistical analysis

Principal component analysis based on the presence/absence of physiological characteristics was done in Canoco 5.0^46^ to visualize the grouping of strains from one year old and three year old plants. Differences between numbers of strains from one year old and three year old plants with antimicrobial activity were tested with Fisher’s exact test. Germination percentages were transformed using centered log ratio transformation (clr), and tested using one-way analysis of variance (ANOVA) and Tukey’s post hoc test. Plant properties and inhibition zone data were analyzed using ANOVA. Significant differences between means were compared using Duncan’s multiple range test at p < 0.05. The results were expressed as mean ± SD. Statistical analyses were performed using the SPSS version 20.0 software package for Windows, R statistical software^47^, and package compositions in R^48^.

## Results

### Isolation and identification of strains

Altogether we isolated 36 and 52 strains that showed morphological characteristics of actinobacteria from one year old and three year old *G*. *inflata* plants, respectively. Most of the strains were isolated from roots (n_1Y_ = 17; n_3Y_ = 25) followed by stem (n_1Y_ = 8; n_3Y_ = 12), leaf (n_1Y_ = 8; n_3Y_ = 10), and only three and five strains were isolated from bark of one year old and three year old liquorice plants, respectively.

**Figure 1:**
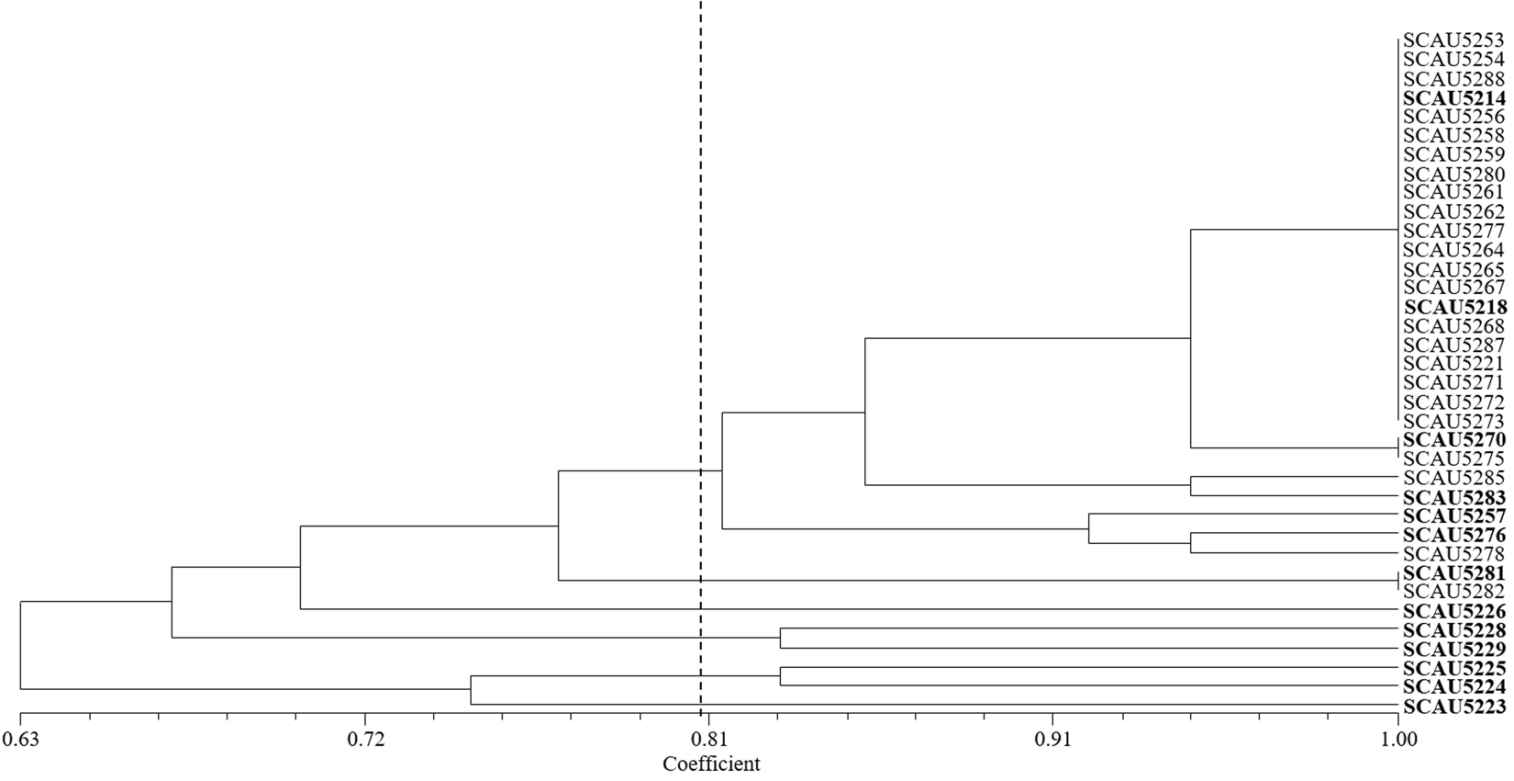
UPGMA dendrogram based on the 16S r DNA PCR-RFLP fingerprints of strains isolated from one year old *Glycyrrhiza inflata* Bat.

**Figure 2:**
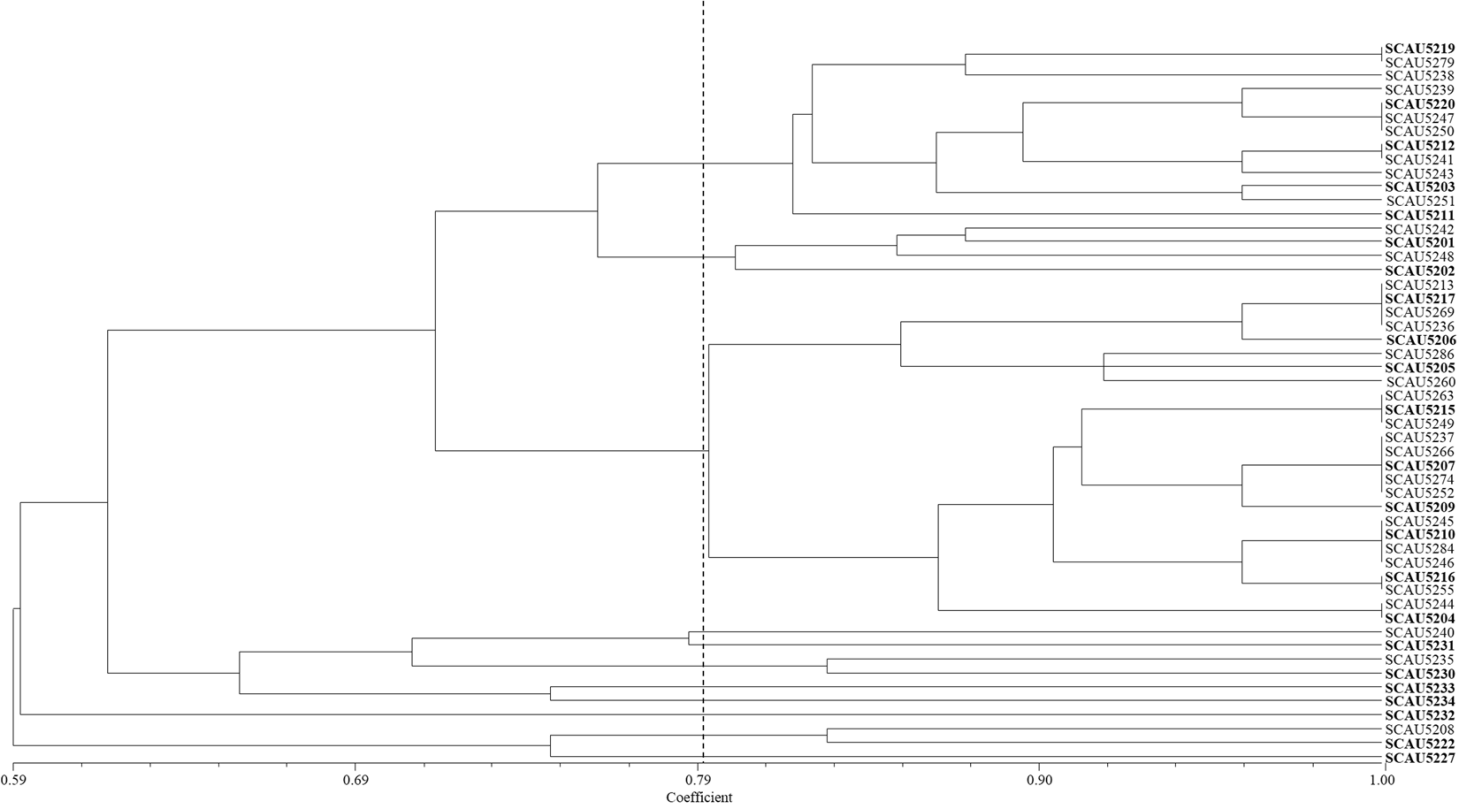
UPGMA dendrogram based on the 16S r DNA PCR-RFLP fingerprints of strains isolated from three year old *Glycyrrhiza inflata* Bat.

The 36 strains isolated from one year old liquorice plants were assigned to six groups at 80% similarity level in the RFLP analysis (Fig. 1). The isolates formed one dominant group of 28 strains that were further separated into subgroups. The other five groups contained 1–2 strains. The 52 strains from three year old plants were assigned to ten groups at 80% similarity level (Fig. 2). The biggest groups contained 25 and 13 strains that were further divided into subgroups. The other eight groups contained 1–4 strains. The RFLP fingerprints of the strains from one year old plants were not detected among those from three year old plants and *vice versa*. Based on the RFLP, one to thirteen representative strains per group were selected for subsequent 16S rRNA gene sequencing and physiological analyses.

**Figure 3:**
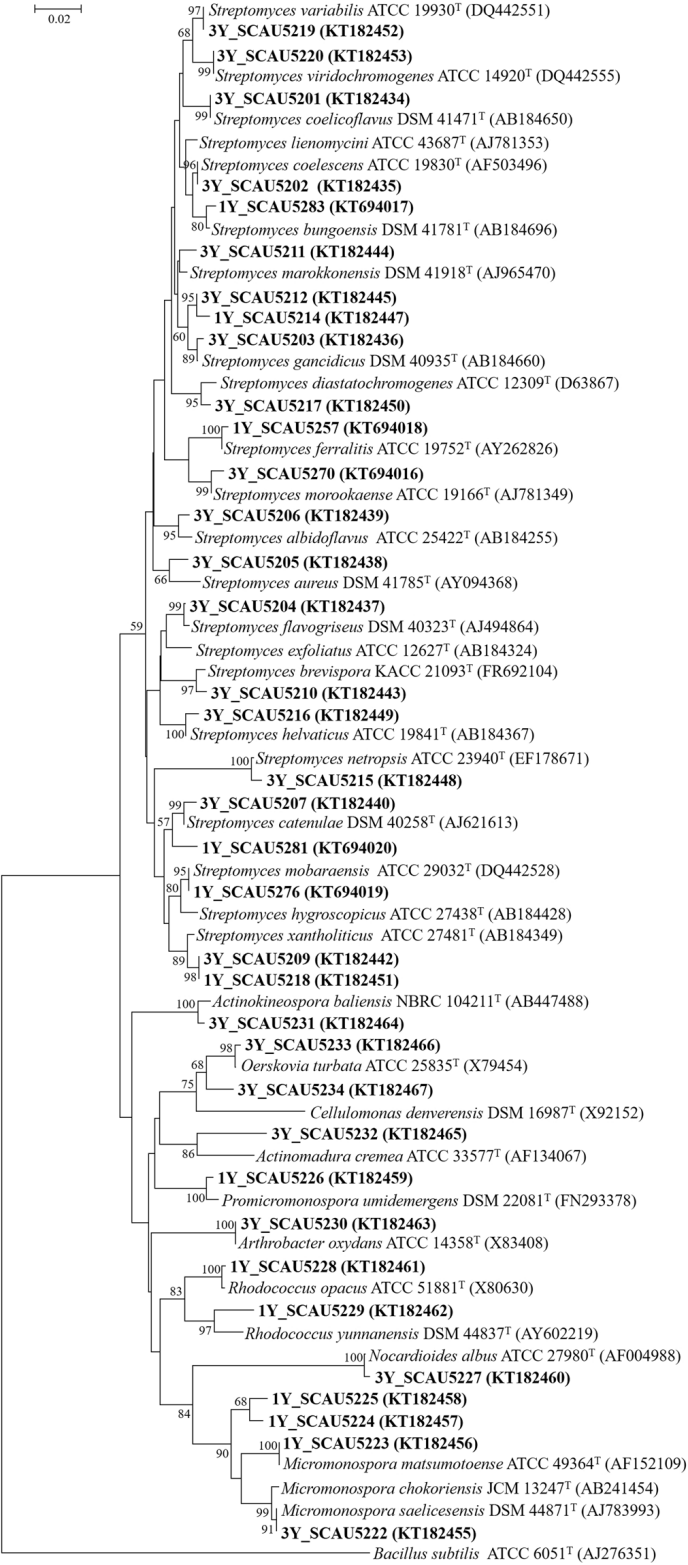
Neighbour-joining tree based on 16S rDNA sequences of actinobacteria closely associated with *Glycyrrhiza inflata* Bat. The numbers at the nodes indicate the level of boot strap support (%) based on 1000 resamplings; only values above 50% are given. The scale bar corresponds to 0.02 substitutions per nucleotide position. Numbers in parentheses are the NCBI GenBank accession numbers. The strains isolated in this study are highlighted in bold.

The 16S rRNA gene sequences of 13 representative strains from one year old liquorice plants were aligned with the 99.1–100% similar sequences of type strains retrieved from databases (Table 1). The strains belonged to the orders Streptomycetales, Corynebacteriales, Micromonosporales, and Micrococcales. Most of them belonged to genus *Streptomyces* (Table 1 and Fig. 3). *Streptomyces*, *Micromonospora*, and *Rhodococcus* strains were found in both root and stem, *Streptomyces* and *Promicromonospora* in leaf, and *Streptomyces* in fruit (Table 1).

**Table 1:** Actinobacteria strains isolated from *Glycyrrhiza inflata* Bat., and the closest type strains based on 16S rRNA gene sequence similarity.

Source	Strain	Genbank No.	Order	Closest type strain	Similarity (%)
**One year old** ***G***. ***inflata***					
Root	SCAU5214	KT182447	Streptomycetales	*Streptomyces thinghirensis* DSM 41919^T^ (FM202482)	99.7
Bark	SCAU5218	KT182451	Streptomycetales	*Streptomyces xantholiticus* ATCC 27481^T^ (AB184349)	99.4
Root	SCAU5223	KT182456	Micromonosporales	*Micromonospora matsumotoense* ATCC 49364^T^ (AF152109)	99.9
Stem	SCAU5224	KT182457	Micromonosporales	*Micromonospora chalcea* DSM 43026^T^ (X92594)	99.1
Root	SCAU5225	KT182458	Micromonosporales	*Micromonospora chalcea* DSM 43026^T^ (X92594)	99.9
Leaf	SCAU5226	KT182459	Micrococcales	*Promicromonospora umidemergens* DSM 22081^T^ (FN293378)	99.1
Stem	SCAU5228	KT182461	Corynebacteriales	*Rhodococcus opacus* ATCC 51881^T^ (X80630)	99.2
Root	SCAU5229	KT182462	Corynebacteriales	*Rhodococcus cerastii* LMG 26203^T^ (FR714842)	100
Stem	SCAU5257	KT694016	Streptomycetales	*Streptomyces ferralitis* ATCC 19752^T^ (AY262826)	99.5
Bark	SCAU5270	KT694016	Streptomycetales	*Streptomyces morookaense* ATCC 19166^T^ (AJ781349)	99.7
Root	SCAU5276	KT694019	Streptomycetales	*Streptomyces mobaraensis* ATCC 29032^T^ (DQ442528)	99.8
Leaf	SCAU5281	KT694020	Streptomycetales	*Streptomyces decoyicus* DSM 41427^T^ (EU170127)	100
Root	SCAU5283	KT694017	Streptomycetales	*Streptomyces bungoensis* DSM 41781^T^ (AB184696)	99.5
**Three year old** ***G***. ***inflata***					
Root	SCAU5201	KT182434	Streptomycetales	*Streptomyces coelicoflavus* DSM 41471^T^ (AB184650)	100
Leaf	SCAU5202	KT182435	Streptomycetales	*Streptomyces coelescens* ATCC 19830^T^ (AF503496)	100
Root	SCAU5203	KT182436	Streptomycetales	*Streptomyces gancidicus* DSM 40935^T^ (AB184660)	99.7
Stem	SCAU5204	KT182437	Streptomycetales	*Streptomyces flavogriseus* DSM 40323^T^ (AJ494864)	99.8
Root	SCAU5205	KT182438	Streptomycetales	*Streptomyces rhizosphaerihabitans* KACC 17181^T^ (HQ267983)	98.7
Bark	SCAU5206	KT182439	Streptomycetales	*Streptomyces albidoflavus* ATCC 25422^T^ (AB184255)	99.3
Stem	SCAU5207	KT182440	Streptomycetales	*Streptomyces catenulae* DSM 40258^T^ (AJ621613)	99.7
Leaf	SCAU5209	KT182442	Streptomycetales	*Streptomyces xantholiticus* ATCC 27481^T^ (AB184349)	99.4
Leaf	SCAU5210	KT182443	Streptomycetales	*Streptomyces brevispora* KACC 21093^T^ (FR692104)	99.5
Leaf	SCAU5211	KT182444	Streptomycetales	*Streptomyces marokkonensis* DSM 41918^T^ (AJ965470)	99
Leaf	SCAU5212	KT182445	Streptomycetales	*Streptomyces lienomycini* ATCC 43687^T^ (AJ781353)	98.5
Root	SCAU5215	KT182448	Streptomycetales	*Streptomyces netropsis* ATCC 23940^T^ (EF178671)	99.7
Bark	SCAU5216	KT182449	Streptomycetales	*Streptomyces helvaticus* ATCC 19841^T^ (AB184367)	99.7
Root	SCAU5217	KT182450	Streptomycetales	*Streptomyces diastatochromogenes* ATCC 12309^T^ (D63867)	99.3
Stem	SCAU5219	KT182452	Streptomycetales	*Streptomyces variabilis* ATCC 19930^T^ (DQ442551)	100
Leaf	SCAU5220	KT182453	Streptomycetales	*Streptomyces viridochromogenes* ATCC 14920^T^ (DQ442555)	100
Root	SCAU5222	KT182455	Micromonosporales	*Micromonospora saelicesensis* DSM 44871^T^ (AJ783993)	100
Root	SCAU5227	KT182460	Propionibacteriales	*Nocardioides albus* ATCC 27980^T^ (AF004988)	99.7
Leaf	SCAU5230	KT182463	Micrococcales	*Arthrobacter oxydans* ATCC 14358^T^ (X83408)	100
Root	SCAU5231	KT182464	Micromonosporales	*Actinokineospora baliensis* NBRC 104211^T^ (AB447488)	99.5
Root	SCAU5232	KT182465	Streptosporangiales	*Actinomadura cremea* ATCC 33577^T^ (AF134067)	95.7
Stem	SCAU5233	KT182466	Micrococcales	*Oerskovia turbata* ATCC 25835^T^ (X79454)	99.5
Root	SCAU5234	KT182467	Micrococcales	*Cellulomonas pakistanensis* DSM 24792^T^ (AB618146)	98.5

The 23 representative strains from three year old plants belonged to Streptomycetales, Micromonosporales, Micrococcales, Propionibacteriales, and Streptosporangiales with 98.5–100% similarity to the closest matching type strains (Table 1). The strains were more diverse than those from one year old plants, and represented ten genera: *Streptomyces*, *Micromonospora*, *Actinokineospora*, *Arthrobacter*, *Actinomadura*, *Oerskovia*, *Cellulomonas*, *Nocardioides*, *Promicromonospora*, and *Rhodococcus* (Fig. 3). Strains belonging to six genera were isolated from root (Table 1). *Streptomyces* strains were isolated from all organs, *Actinokineospora* strains were isolated from both root and stem, and an *Arthrobacter* strain from leaf.

To estimate if the isolated strains were representative of the actinobacterial diversity in *G*. *inflata*, twenty DGGE bands were excised for sequencing. The sequences were affiliated with nineteen genera, out of which four were identified among the isolated strains, suggesting that the isolation methods had captured less than half of the endophytic genera (Table 2).

**Table 2:** Identification and distribution of actinobacteria excised and sequenced from DGGE bands derived from *Glycyrrhiza inflata* Bat.

DGGE band	Distribution of bands in DGGE profile								Phylogenetic group (Order)	Closest relative sequence in Genbank (Genus)	Similarity (%)	Accession number
	R1	R3	S1	S3	L1	L3	B1	B3				
LG7	▲	∇	▲	∇	∇	∇	∇	∇	Actinomycetales	Uncultured actinobacterium (AY177764)	99.0	MF375034
LG12	∇	∇	∇	∇	∇	∇	▲	∇	Corynebacteriales	*Gordonia terrae* (KT072092)	99.0	MF375039
LG9	∇	∇	∇	∇	▲	▲	∇	∇		*Mycobacterium aubagnense* (KR995240)	99.5	MF375036
LG13	▲	∇	▲	∇	▲	∇	▲	∇		*Rhodococcus artemisiae* (NR_108785)	99.4	MF375040
LG5	∇	∇	▲	∇	▲	∇	▲	∇		*Nocardia cyriacigeorgica* (LC055493)	98.9	MF375032
LG1	∇	∇	∇	▲	∇	▲	▲	∇	Geodermatophilales	*Blastococcus* sp.(JX949617)	100.0	MF375028
LG18	▲	∇	∇	∇	∇	∇	∇	∇		*Geodermatophilus* sp.(KC793204)	98.1	MF375045
LG3	∇	▲	∇	▲	∇	∇	▲	∇		*Citricoccus* sp. (KM376500)	99.5	MF375030
LG11	▲	▲	▲	▲	▲	∇	∇	∇		*Microbacterium oxydans* (KP282728)	100.0	MF375038
LG4	▲	▲	▲	▲	▲	▲	▲	▲	Micromonosporales	*Micromonospora saelicesensis* (KT200431)	99.5	MF375031
LG17	∇	∇	▲	▲	▲	▲	∇	∇		*Jishengella endophytica* (KP209418)	98.4	MF375044
LG2	▲	▲	∇	∇	∇	∇	∇	∇	Nakamurellales	*Nakamurella panacisegetis* (NR_108869)	99.5	MF375029
LG10	∇	▲	∇	▲	∇	∇	▲	∇	Propionibacteriales	*Kribbella swartbergensis* (KP052783)	99.0	MF375037
LG20	∇	▲	∇	▲	∇	▲	∇	▲		*Nocardioides dubius* (NR_043280)	98.6	MF375047
LG6	∇	▲	∇	▲	∇	∇	∇	∇	Pseudonocardiales	*Prauserella sediminis* (NR_116674)	100.0	MF375033
LG15	∇	▲	∇	▲	∇	▲	▲	▲		*Pseudonocardia* sp. (LN614620)	98.5	MF375042
LG16	▲	▲	▲	▲	▲	▲	▲	▲		*Saccharopolyspora* sp. (KF673492)	99.5	MF375043
LG8	▲	▲	▲	∇	∇	∇	∇	∇	Streptomycetales	*Streptomyces fradiae* (KC834606)	100.0	MF375035
LG19	∇	▲	∇	▲	∇	▲	∇	∇		*Streptomyces pactum* (KP209436)	98.9	MF375046
LG14	∇	▲	∇	▲	∇	▲	∇	▲	Streptosporangiales	*Nocardiopsis dassonvillei* (KP282801)	98.5	MF375041

^▲^Detected; ^∇^Not detected. R, root; S, stem; L, leaf; B, bark; 1, one year old plant; 3, three year old plant.

### Physiological characteristics of the strains

To further characterize the representative strains, their plant growth promoting (PGP) activity and salt tolerance were tested (Table 3). Nine out of thirteen (69.2%) and fifteen out of 23 (65.2%) strains isolated from one year and three year old plants, respectively, produced IAA at levels ranging from 11.3–71.8 mg L^−1^ and 2.3–46.2 mg L^−1^. SCAU5283 (71.8 mg L^−1^) and SCAU5215 (46.2 mg L^−1^) produced the highest amount of IAA among strains isolated from one year and three year old plants, respectively. Five (38%) and thirteen (56%) strains from one year and three year old plants, respectively, produced siderophores in an iron-deficient culture medium. Two (15.4%) and six (26.1%) strains isolated from one year and three year old plants, respectively, showed a clear halo zone around colony on Pikovskaya’s medium, indicating phosphate solubilization ability. Three (23.1%) and nine (39.1%) strains from one year and three year old plants, respectively, produced chitinase.

**Table 3:** Salt tolerance, antimicrobial activities and plant growth promoting properties of actinobacteria strains isolated from *Glycyrrhiza inflata* Bat.

Strain	NaCl tolerance (mM)				Inhibition of indicator organisms^2^ (mm)							IAA (mg L^−1^)	Siderophore	P solubilization	Chitinase
	200	300	400	500	1	2	3	4	5	6	7				
**One year old** ***G.*** ***inflata***															
*Streptomyces* SCAU5214	*^1^	*	*	—	3.5 ± 0.31^FGH^	—	—	3.1 ± 0.21^H^	—	2.7 ± 0.15^FG^	2.8 ± 0.26^F^	11.5 ± 0.33^J^	*	—	—
*Streptomyces* SCAU5218	*	*	—	—	2.2 ± 0.32^J^	2.3 ± 0.25^EFG^	3.3 ± 0.23^E^	4.2 ± 0.30^F^	—	3.2 ± 0.25^EF^	—	36.7 ± 0.36^D^	—	—	—
*Micromonospora* SCAU5223	—	—	—	—	—	—	—	—	—	—	—	—	—	—	—
*Micromonospora* SCAU5224	*	*	—	—	—	—	—	—	—	—	—	11.3 ± 0.24^J^	—	—	—
*Micromonospora* SCAU5225	—	—	—	—	—	—	—	—	—	—	—	—	—	—	—
*Promicromonospora* SCAU5226	*	—	—	—	5.7 ± 0.41^D^	—	—	—	—	—	—	—	—	—	—
*Rhodococcus* SCAU5228	—	—	—	—	—	—	—	—	—	—	5.9 ± 0.25^D^	—	—	—	—
*Rhodococcus* SCAU5229	*	*	—	—	—	—	—	—	—	—	—	18.2 ± 0.65^G^	—	—	—
*Streptomyces* SCAU5257	*	—	—	—	3.2 ± 0.21^GH^	2.4 ± 0.32^EFG^	6.9 ± 0.25^B^	7.0 ± 0.10^E^	—	—	3.5 ± 0.45^E^	5.4 ± 0.31^M^	—	—	—
*Streptomyces* SCAU5270	*	—	—	—	—	3.6 ± 0.12 ^C^	2.7 ± 0.15^F^	2.6 ± 0.06^I^	2.8 ± 0.15^G^	5.8 ± 0.35^D^	6.5 ± 0.35^C^	27.5 ± 0.55^E^	*	—	*
*Streptomyces* SCAU5276	*	*	*	—	—	—	2.3 ± 0.20^GH^	3.3 ± 0.25^GH^	—	—	—	55.5 ± 0.38^B^	*	*	*
*Streptomyces* SCAU5281	—	—	—	—	—	1.9 ± 0.06^GHI^	1.7 ± 0.25^I^	4.2 ± 0.25^F^	3.4 ± 0.15^F^	5.6 ± 0.25^D^	8.2 ± 0.30^B^	23.9 ± 0.37^F^	*	—	*
*Streptomyces* SCAU5283	*	*	*	*	4.4 ± 0.21^E^	1.8 ± 0.06^GH^	4.1 ± 0.12^C^	3.6 ± 0.16^G^	—	3.6 ± 0.20^E^	—	71.8 ± 0.21^A^	*	*	—
**Three year old** ***G.*** ***inflata***															
*Streptomyces* SCAU5201	*	*	*	*	7.8 ± 0.20^B^	5.5 ± 0.41^B^	3.5 ± 0.06^DE^	12.7 ± 0.21^A^	7.1 ± 0.25^C^	13.5 ± 0.40^A^	4.8 ± 0.21^D^	37.1 ± 0.78^D^	*	*	*
*Streptomyces* SCAU5202	*	*	*		6.5 ± 0.38^C^	2.3 ± 0.12^EFGH^	4.4 ± 0.32^C^	8.3 ± 0.32^C^	8.3 ± 0.26^B^	8.5 ± 0.36^B^	5.5 ± 0.26^D^	6.2 ± 0.48^ML^	*	—	*
*Streptomyces* SCAU5203	*	*	—	—	—	2.9 ± 0.10^D^	4.2 ± 0.15^C^	7.8 ± 0.20^D^	—	—	2.8 ± 0.15^F^	5.7 ± 0.19^M^	*	—	—
*Streptomyces* SCAU5204	*	*	—	—	—	—	—	—	—	—	—	3.5 ± 0.42°	*	—	—
*Streptomyces* SCAU5205	*	*	*	—	3.7 ± 0.32^FG^	—	—	3.2 ± 0.15^H^	4.3 ± 0.26^E^	—	2.2 ± 0.12^G^	18.3 ± 0.23^G^	*	—	*
*Streptomyces* SCAU5206	*	*	—	—	2.7 ± 0.06^IJ^	3.9 ± 0.35^C^	3.3 ± 0.15^E^	8.3 ± 0.31^C^	6.6 ± 0.31^D^	—	—	—	*	—	—
*Streptomyces* SCAU5207	*	*	*	—	—	2.0 ± 0.06^FGH^	3.3 ± 0.20^E^	7.7 ± 0.21^D^	—	—	—	—	*	—	*
*Streptomyces* SCAU5209	*	—	—	—	—	—	2.6 ± 0.15^FG^	8.4 ± 0.31^B^	—	7.0 ± 0.21^C^	3.4 ± 0.10^E^	—	*	—	*
*Streptomyces* SCAU5210	*	*	*	*	6.3 ± 0.40^C^	2.4 ± 0.25^EF^	8.7 ± 0.25^A^	8.4 ± 0.25^C^	—	—	15.5 ± 0.32^A^	—	*	*	—
*Streptomyces* SCAU5211	*	*	—	—	—	2.7 ± 0.21^DE^	3.8 ± 0.06^D^	—	—	—	—	10.7 ± 0.67^K^	—	*	*
*Streptomyces* SCAU5212	*	*	—	—	2.33 ± 0.35^J^	3.6 ± 0.25 ^C^	2.8 ± 0.21^F^	—	3.6 ± 0.26^F^	3.3 ± 0.25^EF^	3.6 ± 0.25^E^	4.9 ± 0.56^MN^	*	*	—
*Streptomyces* SCAU5215	*	*	*	—	8.8 ± 0.32^A^	2.5 ± 0.35^DEF^	2.1 ± 0.10^H^	—	9.5 ± 0.32^A^	—	2.3 ± 0.15^G^	46.2 ± 0.12^C^	*	*	*
*Streptomyces* SCAU5216	*	*	—	—	—	3.5 ± 0.35^C^	2.6 ± 0.15^FG^	—	—	—	—	16.6 ± 0.57^H^	—	*	—
*Streptomyces* SCAU5217	*	—	—	—	3.3 ± 0.17^GH^	2.2 ± 0.12^FGH^	2.3 ± 0.10^GH^	—	—	—	—	9.5 ± 0.36^K^	—	—	—
*Streptomyces* SCAU5219	*	*	*	—	3.9 ± 0.25^EF^	6.3 ± 0.25 ^A^	6.7 ± 0.12^B^	—	8.4 ± 0.15^B^	6.5 ± 0.35^C^	—	2.3 ± 0.32°	*	—	—
*Streptomyces* SCAU5220	—	—	—	—	3.2 ± 0.30^HI^	1.7 ± 0.21^GHI^	3.6 ± 0.26^DE^	—	3.3 ± 0.21^F^	2.5 ± 0.31^G^	2.0 ± 0.30^G^	13.5 ± 0.66^I^	*	—	—
*Micromonospora* SCAU5222	*	—	—	—	4.3 ± 0.29^E^	2.6 ± 0.25^DE^	4.2 ± 0.27^C^	—	—	—	—	7.6 ± 0.71^L^	—	—	*
*Nocardioides* SCAU5227	*	—	—	—	—	—	—	—	—	—	—	—	—	—	—
*Arthrobacter* SCAU5230	—	—	—	—	—	—	—	—	—	—	—	—	—	—	—
*Actinokineospora* SCAU5231	*	—	—	—	1.57 ± 0.12^K^	2.3 ± 0.17E^FGH^	3.6 ± 0.20^DE^	—	—	—	4.4 ± 0.31^D^	9.4 ± 0.57^K^	—	—	—
*Actinomadura* SCAU5232	—	—	—	—	2.5 ± 0.30^J^	—	—	—	—	2.6 ± 0.13^G^	—	—	—	—	*
*Oerskovia* SCAU5233	—	—	—	—	—	—	—	—	—	—	—	—	—	—	—
*Cellulomonas* SCAU5234	—	—	—	—	—	2.1 ± 0.32F^GH^	2.8 ± 0.21^F^	—	—	—	—	19.6 ± 0.54^G^	—	—	—

The values are mean ± standard deviation (n = 3). Different letters in a column indicate statistically significant differences (p < 0.05, Duncan’s multiple range test).

(1) *Growth or PGP activity detected; ^—^No growth, no inhibition, or PGP activity not detected.

(2) Indicator organisms: 1: *Mycogone perniciosa* Magn [SCAU3216]; 2: *Curvularia lunata* Boedijn [SCAU3697]; 3: *Alternaria alternata* (Fries) Keissler [SCAU3471]; 4: *Fusarium graminearum* Sehw. [SCAU3741]; 5: *Fusarium oxysporum* [SCAU3221]; 6: *Staphylococcus aureus* [ATCC 25923]; 7: *Escherichia*. *coli* [ATCC35218].

**Figure 4:**
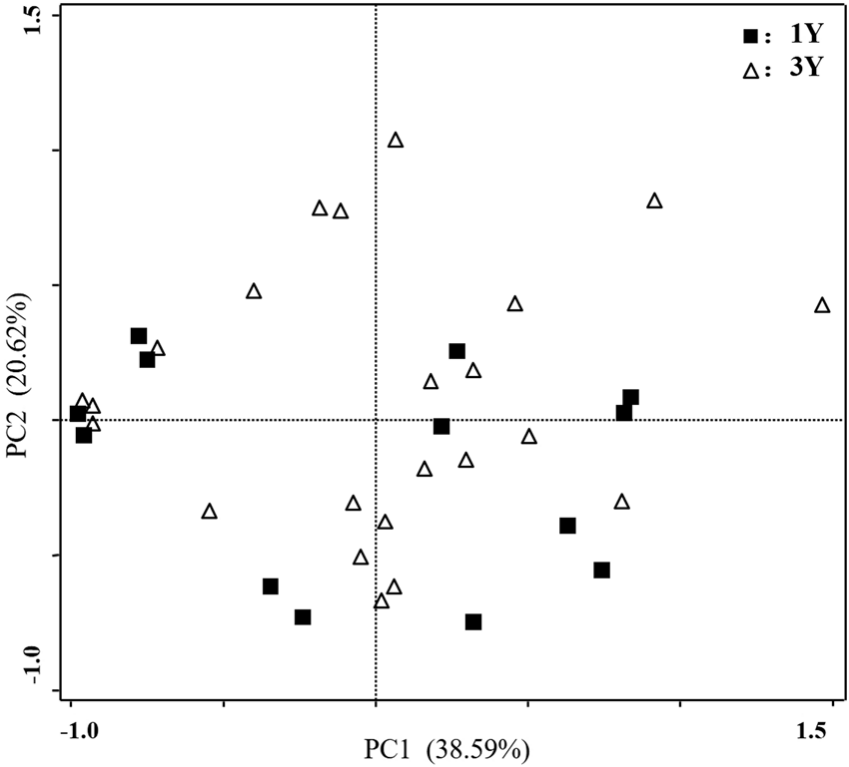
Principal component analysis based on the presence/absence of antimicrobial and PGP activities of actinobacteria strains isolated from one year old (1Y) and three year old (3Y) *Glycyrrhiza inflata* Bat. Only isolates that showed at least one activity are included.

All strains grew in media with 100 mM NaCl. *Streptomyces* strains SCAU5201, SCAU5210, and SCAU5283 tolerated 500 mM NaCl. Nine (69.2%), six (46.2%), and three (23.1%) strains from one year old plants tolerated 200 mM NaCl, 300 mM NaCl, and 400 mM NaCl, respectively. Eighteen (78.3%), thirteen (56.5%) and seven (30.4%) strains from three year old plants tolerated 200 mM NaCl, 300 mM NaCl, and 400 mM NaCl, respectively. In the principal component analysis, the strains from one year old and three year old liquorice plants were not separated based on physiological characteristics (Fig. 4).

### Analysis of antimicrobial activities

The antimicrobial activities of representative strains were tested against seven indicator organisms (Table 3). Differences between numbers of strains with antimicrobial activity from one year old and three year old plants were not statistically significant. Nine out of thirteen (69.2%) strains from one year old plants and twenty out of 23 (86.9%) strains from three year old plants showed antogonistic activity against at least one of the seven indicator organisms.

Altogether 23 out of the 36 strains inhibited the growth of fungus *Alternaria alternate* (Table 3). At the other end, the growth of *Fusarium oxysporum* was inhibited by only ten strains. The growth of bacteria *Staphylococcus aureus* and *Escherichia coli* were inhibited by twelve and fifteen strains, respectively.

All 23 *Streptomyces* strains except SCAU5204 inhibited the growth of at least two indicator organisms (Table 3). *Streptomyces* SCAU5201 and SCAU5202 exhibited broad spectrum antimicrobial activities by inhibiting all the seven indicator organisms. In addition, four other *Streptomyces* strains (SCAU5212, SCAU5220, SCAU5270 and SCAU5281) inhibited the growth of six indicators. Seven out of thirteen rare actinobacteria strains did not inhibit any of the indicator organisms. Out of the rare actinobacteria, *Actinokineospora* SCAU5231 inhibited the widest range of indicator organisms, altogether four.

### The plant growth promotion activity of selected actinobacterial strains

The eight *Streptomyces* strains that grew with 400 mM NaCl and produced indoleacetic acid (IAA) were selected for assessing their effect on *G*. *inflata* seed germination under salt stress. In line with most of the isolates being from roots, six of the strains were from roots, and the other two from leaf and stem. The seed germination rate decreased with the increasing NaCl concentration (Table 4). The higher the NaCl concentration, the more there were strains that did not differ from the non–inoculated control treatment. At 400 mM NaCl, the germination rate of the seeds inoculated with *Streptomyces* SCAU5283 were the highest (Table 4). Compared to the non–inoculated treatment, strains SCAU5201, SCAU5207, SCAU5276, and SCAU5283 increased the seed germination rate under all NaCl concentrations tested.

**Table 4:** Germination rates of un–inoculated and inoculated *Glycyrrhiza inflata* Bat. seeds at different NaCl concentrations. The inoculants were *Streptomyces* sp. strains isolated from *G. inflata*. The values are percentage ± standard deviation (n = 3). Different superscript letters in a column indicate statistically significant differences (p < 0.05, Tukey’s post hoc test).

Inoculant	NaCl concentration				
	0 mM	100 mM	200 mM	300 mM	400 mM
Control	22.0 ± 1.0^F^	14.7 ± 0.6^EF^	12.7 ± 1.2^E^	10.0 ± 1.0^E^	6.0 ± 1.0^C^
SCAU5214	24.3 ± 0.6^CD^	16.0 ± 1.0^E^	14.0 ± 1.0^DE^	11.7 ± 0.6^D^	7.3 ± 1.2^C^
SCAU5283	28.0 ± 1.0^A^	25.3 ± 1.2^A^	23.3 ± 0.6^A^	15.7 ± 1.2^AB^	11.7 ± 0.6^A^
SCAU5276	26.7 ± 0.6^AB^	19.0 ± 1.0^D^	17.7 ± 0.6^B^	14.7 ± 1.5^BC^	9.0 ± 0.0^B^
SCAU5219	23.7 ± 0.6^DE^	13.3 ± 0.6^F^	16.0 ± 1.0^C^	9.7 ± 0.6^E^	6.0 ± 1.0^C^
SCAU5201	25.7 ± 0.6^BC^	22.0 ± 1.0^BC^	17.7 ± 0.6^B^	13.3 ± 0.6^CD^	9.7 ± 0.6^B^
SCAU5202	24.7 ± 1.2^CD^	21.7 ± 0.6^C^	15.3 ± 0.6^CD^	11.7 ± 0.6^D^	6.3 ± 0.6^C^
SCAU5205	22.7 ± 1.5^EF^	19.0 ± 1.0^D^	15.0 ± 1.0^CD^	13.0 ± 1.0^D^	6.7 ± 0.6^C^
SCAU5215	26.7 ± 0.6^AB^	23.3 ± 0.6^B^	18.0 ± 0.0^B^	16.3 ± 0.6^A^	10.3 ± 0.6^B^

**Figure 5:**
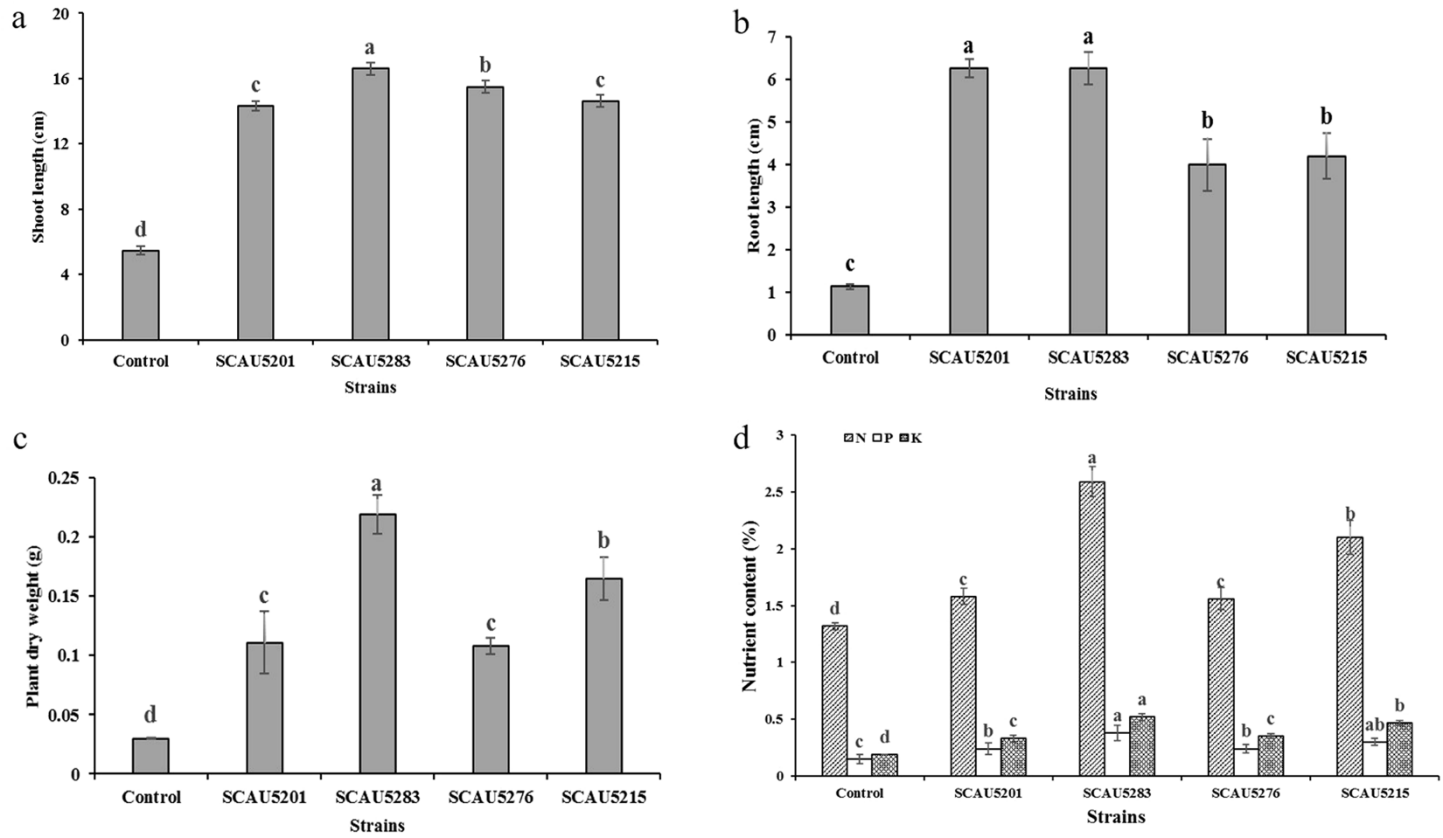
The shoot length (**a**), root length (**b**), plant dry weight (**c**), and nutrient contents (**d**) of *Glycyrrhiza inflata* Bat. inoculated with *Streptomyces* sp. strains. Control = un-inoculated *Glycyrrhiza inflata* Bat. The values are mean ± standard deviation (n = 3). Different superscript letters on a column indicate statistically significant differences (p < 0.05, Duncan’s multiple range test).

The above mentioned four strains were selected for assessing their effect of *G*. *inflate* seedling growth in a greenhouse experiment. Compared to the non–inoculated treatment, all the four strains increased plant shoot length, root length, dry weight, and N, P and K contents significantly (Fig. 5). All the measured parameters were greatest in plants inoculated with strain SCAU5283.

## Discussion

Liquorice is known as “the king of Chinese medicine” that is widely applied in pharmaceutical and food industry due to its medicinal value and sweet taste. The liquorice plants (*Glycyrrhiza* spp.) tolerate harsh environmental conditions, and they may be applied for example in reclaiming saline soils^20^. *Glycyrrhiza* spp. are nodulated by rhizobial bacteria that fix atmospheric nitrogen and thus promote the growth of the host plant^17–19^. Like numerous plant species^5^, *Glycyrrhiza* spp. host endophytic actinobacteria^23^. Many endophytic bacteria have plant growth promoting (PGP) ability, and they can increase germination and growth of their host plants under environmental stress^8,9,21^.

Since the endophytic communities change over time^24,49,50^, sampling plants at different growth stages may increase possibilities to isolate strains with desired characteristics. We isolated actinobacteria from the roots, stems, leaves and bark of one year old and mature three years old liquorice plants, and tested their PGP and antimicrobial activities. In line with the observation that endophytic bacteria enter through roots and then migrate to other organs, most of the strains were isolated from roots. In addition to the genera *Streptomyces*, *Micromonospora*, and *Rhodococcus* isolated in our previous study^23^, in this study seven genera more were isolated from *G*. *inflata*. Most of them have been previously reported as endophytes of medicinal or other plants^25,51–54^. *Actinokineospora* spp. have been isolated from soil, plant litter and sponges^55,56^, but, to our knowledge, not from inside a plant. More diversity was revealed by DGGE, highlighting the need to develop cultivation methods to isolate rare actinobacteria species for assessing their PGP and antimicrobial activities.

The actinobacteria closely associated with plants have a long-held relationship with host plants, and they may play an active role in plant development and also protect the hosts against pathogens^57,58^. In our work, we assessed four PGP characteristics: production of indole acetic acid (IAA), siderophore, chitinase, and phosphate solubilization activities. All *Streptomyces* strains showed at least one activity, whereas over half of the rare actinobacteria strains did not show any. IAA is a plant growth promoting hormone, produced not only by plants themselves but also by many plants associated bacteria. As in earlier studies^6,8,59^, most of the IAA-producing strains belonged to genus *Streptomyces*. Siderophores chelate Fe (III), and siderophores secreted by actinobacteria contribute to plant protection by competing with potential pathogens for iron^60^. Many *Streptomyces* spp. produce siderophores^61,62^, and in our study all of the siderophore producing strains were affiliated with *Streptomyces*.

Phosphorus is one of the most important nutrients for plant growth and development. Phosphate solubilizing bacteria are effective in releasing P through solubilization and mineralization, and have been used as inoculants to improve the growth and yield of crop plants. A considerable number of bacterial species associated with plant rhizosphere have a high capacity in solubilizing P^63^. Among the endophytes, 19% of isolates from the medicinal plant *Ferula songorica*, half of the actinobacteria strains from seven medicinal plant species, and four out of nineteen isolates from *Jatropha curcas* solubilized P^6,59,64^. In our work, the proportion of P solubilizing strains was within the same range: eight *Streptomyces* strains solubilized P.

Actinobacteria isolated from various plant tissues inhibited pathogens by producing active compounds and chitinase^65^. Endophytic actinobacteria that produced chitinase protected plants against phytopathogenic fungi^66^. Endophytes with chitinase activity suppressed fungal pathogens by degrading cell wall and thus bursting spores and hyphal tips, thereby inhibiting spore germination and germ tube elongation^32^. In our study, all the strains with chitinase activity were able to inhibit pathogens. However, most of the antifungal strains did not produce chitinase, suggesting that those strains have alternative mechanisms to inhibit the growth of fungi.

Actinobacteria closely associated with terrestrial and marine plants are considered vital sources of secondary metabolites with potential antimicrobial activity^67,68^. Similar with our previous research^23,25^, almost all of the *Streptomyces* strains showed antimicrobial activity against at least one of the tested indicator organisms. In addition, some of the *Actinokineospora*, *Cellulomonas*, *Actinomadura*, *Nocardioides*, and *Rhodococcus* strains inhibited the growth of indicator organisms, indicating that rare actinobacteria are a potent storehouse that should not be ignored when searching for natural products.

In general, the *Streptomyces* strains tolerated higher concentrations of NaCl and inhibited the growth of greater number of indicator organisms than the rare actinobacteria. However, it should be noted that the difference between Streptomyces and rare actinobacteria *in vitro* does not necessarily indicate a difference *in vivo*. *Streptomyces* strains are relatively easier to cultivate than the rare actinobacteria^69^. Possibly the PGP, salt tolerance, and antimicrobial activities of the *Streptomyces* strains are also more strongly expressed than those of the rare actinobacteria.

Salt tolerant actinobacteria with plant growth promoting as well as antagonistic activity against pathogens could alleviate the deleterious effect of salinity^6,59^. We selected the eight strains that tolerated high level of salt and produced IAA to evaluate if the strains could promote *G*. *inflata* seed germination under salt stress *in vivo*. All the eight strains belonged to genus *Streptomyces*. In the germination assay at 200 mM and higher concentrations of salt, inoculation with the four strains that had produced highest amounts of IAA and solubilized P resulted in highest germination rates. Concluding that the strains affected germination through IAA would require further analyses. Exogenous IAA and IAA producing bacterial strains have increased germination rate under salt stress^21^. However, IAA is not thought to affect germination directly, yet it may interact with gibberellins and ethylene and indirectly affect germination^70^.

Phytohormone producing strains have been proposed to alleviate salt stress and facilitate plant growth in harsh environment^21^. We assayed the effect of the abovementioned four strains on the growth of *G*. *inflata* under salt stress in a greenhouse experiment. The growth of all the inoculated plants was significantly better than that of the un–inoculated plants. The growth promotion *in vivo* was not directly related to the degree of IAA production *in vitro*; strains SCAU5215 and SCAU5201 outperformed SCAU5276 that produced higher amount of IAA. The best promoters of *G*. *inflata* growth, strains SCAU5283 and SCAU5215, inhibited a wide range of indicator organisms, and may thus be considered as promising candidates to be applied in inoculating *G*. *inflata* in reclaiming saline soils.

In summary, the actinobacteria strains isolated from *G*. *inflata* represented ten genera. Most of the strains had plant growth promoting characteristics *in vitro*, tolerated 200 mM NaCl and inhibited the growth of at least one indicator organism. The eight selected *Streptomyces* strains increased the germination rate of *G*. *inflata* seeds under salt stress. In addition, the four best seed germination promoters promoted the growth of *G*. *inflata in vivo*.

## Data Availability

The sequences obtained in this study have been assigned GenBank (National Center for Biotechnology Information, USA) accession numbers KT182434-KT182467, KT694016-KT694020, and MF375028-MF375047 (https://www.ncbi.nlm.nih.gov/genbank/).
